# Pharmacists’ Knowledge, Perception, and Prescribing Practice of Probiotics in the UAE: A Cross-Sectional Study

**DOI:** 10.3390/antibiotics13100967

**Published:** 2024-10-13

**Authors:** Maram O. Abbas, Hanan Ahmed, Eisha Hamid, Dyshania Padayachee, Menah Talla Abdulbadia, Sohila Khalid, Ahmed Abuelhana, Bazigha K. Abdul Rasool

**Affiliations:** 1 Institute of Public Health, College of Medicine & Health Sciences, UAE University, Al Ain 15551, United Arab Emirates; maram.abbas88@gmail.com; 2 Pharmacy Practice Department, Dubai Pharmacy College for Girls, Dubai P.O. Box 19099, United Arab Emirates; 3 Pharmaceutical Sciences Department, Dubai Pharmacy College for Girls, Dubai P.O. Box 19099, United Arab Emirates; hanan12_7@hotmail.com (H.A.); eishahamid5846@gmail.com (E.H.); padayacheedyshania@gmail.com (D.P.); menahmeamo251@gmail.com (M.T.A.); sohilakhalid80@gmail.com (S.K.); 4 School of Pharmacy and Pharmaceutical Sciences, Ulster University, Coleraine BT52 1SA, UK; a.abuelhana@ulster.ac.uk

**Keywords:** probiotics, pharmacists’ knowledge, prescribing practices, UAE, gastrointestinal health, immune support

## Abstract

Background: The human body is a complex and interconnected system where trillions of microorganisms, collectively known as the gut microbiota, coexist with these cells. Besides maintaining digestive health, this relationship also impacts well-being, including immune function, metabolism, and mental health. As frontline healthcare providers, pharmacists are pivotal in promoting the benefits of probiotics for immune support. This study explored pharmacists’ knowledge, perception, and practice behavior in the UAE towards the implication of probiotic application beyond digestive health, such as cardiovascular and mental health impacts and their diverse dosage forms. Method: An online self-administered survey was distributed among pharmacists in the UAE. Data were collected through personal visits to pharmacies, where pharmacists were approached and asked to complete the questionnaire. The sample size included 407 pharmacists, determined using the formula for proportions with a 95% confidence level and a 5% margin of error. Statistical analysis was performed using SPSS version 29. Descriptive statistics were used to summarize demographic characteristics and survey responses. The knowledge levels were categorized into poor, moderate, and good. Chi-square analysis was employed to investigate associations between demographic factors and knowledge levels, with a significance level set at *p* < 0.05, enhancing the robustness of the study’s findings. Results: This study included 407 completed eligible responses. About 63.56% of participants were female, with 52.1% employed in pharmacy chains. While 91.2% of pharmacists recognized probiotics’ role in immune support, only 30% were aware of their cardiovascular benefits. Moreover, chewing gum was the least known dosage form of probiotics, recognized by only 16.7% of respondents. Additionally, only 57% of the participants recognized liposomes as a dosage form. In practice, most pharmacists recommended storing probiotics at room temperature, accounting for 66.6%. The most prevalent misconception encountered in the pharmacy setting was the belief that probiotics are primarily intended for gastrointestinal tract problems, at 79.1% of the respondents. Regarding perception, the agreement was observed regarding the safety of probiotics for all ages. Perceived barriers included the high cost of probiotics, with the majority (86.5%) indicating this as a significant obstacle, while lack of demand was identified as the minor barrier by 64.6%. Additionally, an association was found at a significance level of *p* < 0.05 with knowledge, gender, educational level, type and location of pharmacy, and source of information. Conclusions: The study highlights knowledge gaps in pharmacists’ understanding of probiotic applications beyond digestive health, particularly cardiovascular health and depression. Targeted educational interventions are necessary to address these gaps. The findings underscore the importance of ongoing professional development for pharmacists, enhancing their role in patient education and the promotion of probiotics for overall health.

## 1. Introduction

The World Health Organization (WHO) defines probiotics as “live microorganisms which confer a health benefit on the host when administered in adequate amounts” [1]. Since their establishment in 2001, probiotics, including strains like Lactobacillus and Bifidobacterium, have been extensively studied for their impact on human health, particularly their role in modulating the gut microbiota [2,3]. They primarily exert their effects by colonizing the gut and modulating its microbial composition, promoting a favorable balance of beneficial bacteria; this intricate interplay between probiotics and the gut microbiota underscores their crucial role in human health [4,5].

Probiotics have a wide range of therapeutic benefits, impacting many different bodily systems and improving overall health. Moreover, they are known for boosting the immune system and helping the body defend against infections and allergies [6,7,8]. One of the probiotics’ most significant benefits is their role in digestive health. Probiotics help maintain intestinal barrier integrity, regulate bowel movements, and alleviate gastrointestinal disorders such as irritable bowel syndrome, inflammatory bowel disease symptoms, infectious diarrhea, antibiotic-associated diarrhea, and ulcerative colitis [9,10,11]. However, their optimal use presents a challenge due to the lack of clear guidelines, making it difficult for family physicians and patients [12].

Research suggests potential health benefits of probiotics, including anti-obesity, anti-diabetic, and anti-cancer effects, as well as possible COVID-19 neutralization, though these benefits are not yet definitively confirmed [13,14]. In addition, emerging research suggests a link between probiotics and mental health, with evidence indicating their potential to alleviate symptoms of anxiety, depression, and stress through interactions with the gut–brain axis [15,16,17].

A recent meta-analysis has concluded that probiotics significantly reduce total cholesterol and low-density lipoprotein (LDL) cholesterol in individuals diagnosed with hypercholesterolemia [18]. This finding holds particular significance, considering that systemic inflammation is a common characteristic of cardiovascular diseases, which are frequently accompanied by risk factors such as hypertension [19]. The potential of probiotics to influence these interconnected pathways indicates a broader therapeutic potential in managing cardiovascular health [20].

Ensuring probiotic stability for clinical use is crucial, and diverse formulation approaches are actively explored to enhance efficacy through various administration routes [21]. Advances in technology, including microencapsulation and nanotechnology, offer sophisticated delivery systems [22]. While oral forms predominate, evolving technology allows for diverse probiotic dosage forms and administration routes [23]. Recent formulation approaches emphasize diverse dosage forms and technologies to optimize probiotic delivery for enhanced therapeutic effectiveness [24].

The general public and healthcare providers should possess comprehensive knowledge about probiotics’ benefits and proper utilization to ensure optimal usage [25,26]. Since probiotics are readily available without a prescription, individuals need to thoroughly understand their potential advantages and the practical methods for their administration. Pharmacists are crucial in educating patients about probiotics, including their potential benefits for digestive health, immune function, and overall well-being. Furthermore, pharmacists occupy a central position in healthcare provision, serving as frontline healthcare providers with a multifaceted role in patient care [27,28].

Pharmacists should be well-versed in evidence-based indications for probiotic use, appropriate dosage recommendations, and potential safety concerns. Studies conducted in various regions, including Saudi, Pakistan, India, and Nigeria, highlighted the need to raise awareness and enhance healthcare providers’ knowledge of probiotics; these studies found low levels of understanding and awareness among healthcare professionals, including pharmacists, emphasizing the importance of targeted education initiatives [29,30,31,32].

Despite the growing popularity and widespread availability of probiotics in pharmacies, there is a lack of comprehensive information regarding pharmacists’ knowledge levels, perceptions, and practices regarding probiotics in the UAE. Therefore, this study aimed to bridge the knowledge gap by investigating pharmacists’ engagement with probiotics in the healthcare context of the UAE.

## 2. Results

### 2.1. Demographics

A total of 407 responses were collected for this study. Among the participants, 259 (63.65%) were female. Most participants, 262 (64.4%), fell within the age range of 22 to 30. More than half of the participants, 211 (51.8%), completed their higher education in the UAE. Most participants were affiliated with community pharmacies; 212 (52.1%) were part of chain pharmacies, and 82 (20.1%) were independent institutions. The distribution of participants based on the number of prescriptions dispensed per day showed that the majority, 181 (44.5%), reported dispensing 10–19 prescriptions daily (Table 1).

### 2.2. Pharmacist Knowledge Concerning Probiotic Uses and Dosage Forms

Regarding knowledge, 388 (95.3%) knew that probiotics consist of different strains of live bacteria, each offering specific health benefits. Likewise, 390 (95.8%) recognized yogurt as a natural source of beneficial bacteria. The least recognized information about probiotics was their role in alleviating depression symptoms, with only 122 (29.9%) participants being aware of this. In terms of dosage forms, capsules, oral powder, and tablets were the most recognized forms, with recognition rates of 376 (92.4%), 318 (78.1%), and 250 (61.4%), respectively. The least recognized delivery system was nanoparticles, identified by only 173 (42.5%) participants (Table 2).

**Figure 1:**
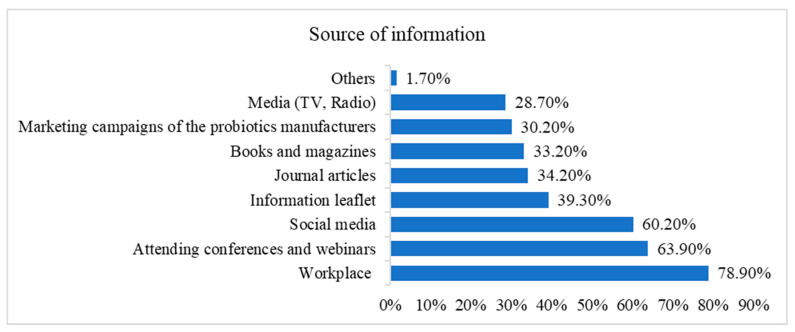
Participants’ sources of information about probiotics.

The participants utilized a variety of information sources. The most used source was the workplace (specifically, the pharmacy setting) selected by 321 (78.9%) respondents. Attending conferences and webinars was also popular, with 260 participants (63.9%) relying on these events for information (Figure 1).

### 2.3. Pharmacist Perception of the Use of Probiotics

**Figure 2:**
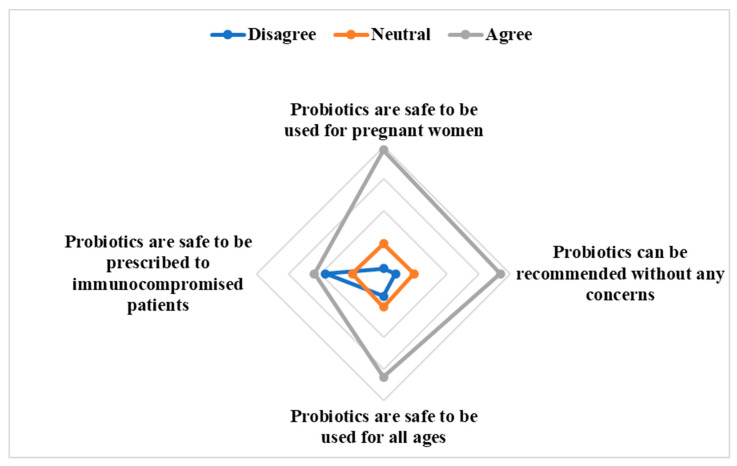
Probiotics safety perception among participants.

Most respondents, 317 (77.9%), believed using probiotics during pregnancy is safe. Additionally, about 262 (64.4%) expressed trust in the safety of probiotics for people of all ages. Interestingly, while participants had high confidence in probiotics’ safety (Figure 2), a considerable portion of 265 (65.1%) believed that the public lacked awareness regarding the benefits and applications of these supplements. Furthermore, an overwhelming majority, 377 (92.6%), showed interest in educational efforts or workshops related to probiotic use (Table 3).

### 2.4. Pharmacist Practice Concerning Probiotic Recommendation and Prescription

There is variation among pharmacists in terms of selling and prescribing probiotics in their pharmacies, with 162 (39.8%) reporting sales 2–3 times a week and 143 (35.1%) reporting daily sales. When prescribing probiotics to boost patients’ immune systems, many pharmacists prescribe them often (179, 44.0%) or always (107, 26.3%). Regarding storage, 271 (66.6%) pharmacists advise room temperature storage. When patients ask about probiotics, most pharmacists prefer to prescribe a suitable probiotic for their specific condition (307, 75.4%) rather than suggesting natural sources or advising them to consult a physician. Some common misconceptions include the belief that probiotics are only for digestive issues (238, 58.5%) or can replace a healthy diet (160 39.3%). Pharmacists frequently prescribe probiotics for gastrointestinal diseases (231, 56.7%). The most requested dosage forms are capsules and tablets (249, 61.2%) (Table 4).

### 2.5. Pharmacists Perceived Barriers to the Optimum Use of Probiotics among the Public

The participants identified several potential barriers to the widespread use of probiotics. The top three perceived concerns were the high cost of the probiotic products (352, 86.5%), the lack of insurance coverage for the probiotic and considering it as a treatment (351, 86.2%), and the lack of advice from doctors (344, 84.5%) (Table 5).

### 2.6. Associations of Knowledge Levels with Demographics and Practice

Most participants, 309 (75.9%), were classified in the moderate knowledge group, followed by the good knowledge group, 71 (17.4%), and the poor knowledge group, 27 (6.6%). Significant associations were found between probiotic knowledge and various demographics. Gender, educational level, type and location of the pharmacy, and source of information all had *p*-values of less than 0.05, indicating their significance (Table 6).

Further regression analysis, as shown in Table 7, revealed that participants with a Doctorate were significantly more likely to have both moderate (OR = 2.98, *p* < 0.001) and good knowledge (OR = 7.07, *p* < 0.001), while those with a Diploma were less likely to have good knowledge (OR = 0.132, *p* = 0.01). Working in Community Chain Pharmacies was also associated with significantly higher odds of good knowledge (OR = 7.411, *p* = 0.004). Other factors, such as gender, age, years of experience, and the number of prescriptions filled per day, were not significantly associated with either moderate or good knowledge levels. The reference (ref.) category for knowledge levels is poor knowledge.

## 3. Discussion

This cross-sectional study investigated pharmacist knowledge, practice, perception, and barriers to probiotics in the UAE. The findings reveal significant knowledge gaps among pharmacists, particularly regarding non-digestive applications of probiotics, and different available dosage forms, which could restrict patient access to the full spectrum of probiotic health benefits. These knowledge deficits are concerning because they may result in missed opportunities to optimize patient care, especially for cardiovascular and mental health conditions, where probiotics have shown promise. The analysis showed that most pharmacists have a moderate level of knowledge about probiotics, and the high cost is a prominent barrier that hinders patient access.

In the current study, most pharmacists firmly understood the fundamental aspects of probiotics. For example, they recognized yogurt as a natural source and understood that probiotics are living microorganisms. However, despite this foundational knowledge, critical gaps remain in understanding the broader clinical applications of probiotics beyond digestive health. A study by Santhanam et al. (2022) on the knowledge of probiotics among dental practitioners produced results consistent with this study’s findings revealing that 70% of participants demonstrated acceptable knowledge levels [33]. However, the finding of Santhanam et al. could not be extrapolated and accurately compared to this study because of the different scope of practice and accessibility between pharmacists and dentists. Interestingly, a global probiotic survey conducted in 30 countries in 2020 involving various healthcare professionals found similar results; about 80% of respondents from each healthcare professional category answered basic knowledge questions correctly. In both this study and the international survey, the use of Lactobacillus acidophilus as a probiotic was well understood, yet the broader therapeutic applications of probiotics were less commonly recognized, suggesting that pharmacists and healthcare professionals may underappreciate the full potential of probiotics on a global scale [30]. Both studies reported high levels of correctness for the use of Lactobacillus acidophilus, with 93.6% accuracy in this study and 92% in the international survey [34]. However, in contrast to these findings, a study conducted in Pakistan in 2021, which assessed healthcare practitioners’ knowledge regarding probiotics, found that only 15.1% of participants demonstrated a comprehensive understanding of probiotics. This highlights significant variations in awareness levels across different regions, emphasizing the need for targeted educational efforts.

However, this study’s findings are concerning as more than half of the participants provided incorrect answers when asked about the specific roles of probiotics, despite the well-established worth of probiotics in respiratory immunity, cardiovascular health, and alleviating depression symptoms [7,35,36]. Several studies have consistently demonstrated improvements in blood pressure, triglycerides, total cholesterol, and LDL cholesterol after probiotic supplementation, highlighting their significant role in enhancing cardiovascular health [37]. Moreover, a systematic review concluded that probiotics show great potential in treating depression [36]. Addressing these knowledge gaps is essential to expanding the use of probiotics beyond gastrointestinal disorders and ensuring that pharmacists can provide comprehensive recommendations, especially for patients at risk of cardiovascular disease or depression.

This study showed that participants with a Doctorate may have greater exposure to advanced education, research, and professional development opportunities, which likely contributes to their higher knowledge levels [38,39]. On the other hand, those with Diplomas may have fewer opportunities for specialized training, impacting their knowledge. Additionally, pharmacists working in community chain pharmacies may receive targeted training or resources related to probiotics, explaining their significantly higher knowledge. Future studies could investigate these potential factors in more detail to better understand and address these disparities.

Regarding gender, although our analysis did not find a significant association between gender and probiotic knowledge, it is possible that societal and professional factors, such as differences in access to continuing education or professional roles, might play a role. While these factors were not directly assessed in our study, future research could explore whether gender-specific educational or professional experiences contribute to variations in knowledge. Understanding these nuances may help in tailoring more effective educational interventions across different demographic groups.

Many studies emphasize the importance of diverse probiotic dosage forms in pharmaceuticals. These varied forms are crucial for optimizing drug delivery, enhancing efficacy and tolerability, and improving patient comfort and convenience [24]. Pharmacists play a pivotal role by knowing the different forms and selecting the most suitable one for each patient’s needs [40,41]. This study examined pharmacists’ awareness of probiotic dosage forms globally. The findings revealed that while many pharmacists were familiar with common forms such as capsules, drops, and tablets, there was less awareness of alternative options like topical gels, creams, sprays, and chewing gum. This gap in knowledge highlights the need for enhanced education on diverse probiotic dosage forms to enable pharmacists to provide more personalized patient care. Additionally, the study evaluated participants’ understanding of various delivery systems used to develop these dosage forms. Our results indicated a significant number of participants expressed confidence in the safety of probiotics. Most believe probiotics are safe for pregnant, immunocompromised, and people of all ages. However, it is essential to note that recent research has revealed potential risks associated with probiotic use, especially among certain vulnerable groups [42]. Regulatory bodies, including the FDA, have specifically issued warnings against the use of probiotics in hospitalized preterm infants due to potential risks of adverse effects. Additionally, some studies highlight concerns about probiotic use in other high-risk populations, such as pregnant women, immunocompromised individuals, and those with structural heart disease. These warnings emphasize the need for caution and individualized assessment when considering probiotic use in these vulnerable groups [43,44,45]. Moreover, the evidence regarding the effectiveness of probiotics in specific conditions affecting pregnant women, such as gestational diabetes mellitus, metabolic syndrome, and preeclampsia, is inconclusive [46]. These findings emphasize the importance of carefully considering probiotic use, particularly in vulnerable populations.

Participants in this study commonly observed a significant deficiency in public awareness about the optimal use of probiotics, a concern supported by existing research. For instance, a 2018 survey conducted by Hiba Barqawi et al. in the UAE revealed that the general public possessed limited knowledge about probiotics [47]. Similarly, research on gastrointestinal patients indicated that most had never used probiotics, and among those who had, the majority relied on products prescribed by healthcare professionals [48]. These findings highlight the urgent need for improved public education on probiotics to foster better understanding and more effective use.

The study revealed that a substantial majority of participants endorse the use of probiotics to boost immune function. Despite the broad evidence supporting the benefits of probiotics for various health conditions, most probiotics dispensed in surveyed pharmacies were aimed at addressing gastrointestinal (GI) issues. This observation is consistent with findings from multiple studies conducted in diverse healthcare settings worldwide, which also underscore the prevalent focus on GI complications in probiotic prescription practices and counseling [49,50].

When it comes to counseling, it is crucial to ensure that patients are appropriately informed about the storage conditions for prescribed medications. However, this study’s results indicate that most pharmacists advise patients to store probiotics at room temperature, which may contradict optimal storage practices. Nevertheless, product labels usually provide specific instructions, and room temperature storage may align with these recommendations. Research by Fenster et al. 2019 highlights the variability in storage requirements among different probiotic products, with some requiring refrigeration during manufacturing [51].

Additionally, recommendations regarding the timing of probiotic consumption, such as taking them before meals, align with research findings suggesting that bacterial survival rates are higher when probiotics are ingested before meals due to slower passage through the stomach [52].

The primary barrier to the general public’s optimal use of probiotics is economic; high costs and lack of insurance coverage for probiotic prescriptions are particularly prominent concerns. This observation is consistent with the survey conducted by Kolady et al. 2018, in which participants of higher-income groups tended to purchase more probiotics than those with lower incomes [53]. Various studies indicated that probiotics may not consistently demonstrate cost-effectiveness, especially in hospitalized patients. However, they have been proven to improve quality-adjusted life years (QALYs) and reduce disability-adjusted life years (DALYs) for specific conditions such as diarrhea [54,55].

Furthermore, in the UAE, probiotic coverage varies depending on specific insurance plans and the type of probiotic supplement. Generally, over-the-counter probiotics are not covered [56]. However, it is essential to note that probiotics are classified as dietary supplements and are not regulated by the United States Food and Drug Administration (FDA). This lack of regulation leads to variability in product quality, purity, and viability.

## 4. Materials and Methods

### 4.1. Study Design

A prospective cross-sectional national survey-based study was conducted between October 2023 and February 2024 to investigate and explore pharmacists’ knowledge, perception, and practice patterns regarding probiotic dosage in the UAE. The survey was formatted as an online, self-administered questionnaire using Google Forms and distributed in person within pharmacy settings across all emirates in the UAE. Pharmacists in diverse pharmacy settings were cordially invited to participate in this study.

### 4.2. Data Collection Sheet

The questionnaire consisted of five sections comprising 20 questions and was investigated to take no more than eight minutes to complete. It was comprised entirely of closed-ended, multiple-selection questions designed to collect quantitative data. It was divided into five distinct sections: Section #1 (Demographics): Collected basic demographic information, including years of experience, pharmacist practice setting, age, and gender; Section #2 (Knowledge): Assessed pharmacists’ knowledge of probiotics, including their benefits, uses, and available formulations; Section #3 (Perception): Gathered insights into pharmacists’ views on the efficacy and safety of probiotics; Section #4 (Practice): Explored how pharmacists integrate probiotics into their clinical practice; and Section #5 (Barriers): Identified challenges pharmacists encounter when prescribing or recommending probiotics. Appendix A.

The items in the questionnaire were adapted from a previous research study and were subjected to a pilot test with a group of thirty-five pharmacists. The reliability of the questionnaire was assessed using Cronbach’s alpha, resulting in a score of 0.9, indicating a high level of internal consistency. Additionally, the questionnaire underwent independent validation by two academic professors and two pharmacy practitioners to ensure its content validity and suitability for the study’s objectives. The survey was administered online using Google Forms. Pharmacists were directly approached in their pharmacies, where they were invited to participate by completing the survey on a provided device.

### 4.3. Sample Size and Sampling

This study focused on pharmacists practicing within the UAE. Inclusion criteria required participants to be actively involved in prescribing and to have direct patient care responsibilities. Pharmacists engaged solely in administrative or compounding roles without patient interaction and those outside the UAE were excluded.

A convenience sampling approach was employed to recruit participants from community and hospital pharmacy settings. This method was chosen due to the logistical challenges of accessing a geographically dispersed population of pharmacists across the UAE. Given the time constraints and the demanding schedules of the pharmacists, convenience sampling was the most efficient and practical method to ensure timely data collection. Data collection was facilitated through personal pharmacy visits and an online self-administered survey. This approach recruited a diverse cohort of pharmacists from various practice settings, enhancing the sample’s representativeness.

The sample size for this study was calculated using the formula for proportions: n = [Z2 * p * (1 − p)]/E2, where n represents the required sample size, Z is the Z-score corresponding to the desired confidence level (e.g., 95% confidence level corresponds to a Z-score of approximately 1.96), p denotes the estimated proportion of pharmacists (utilizing 0.5 for maximum variability due to unavailable estimates), and E signifies the margin of error expressed as a proportion. Assuming a 95% confidence level, a margin of error of 5% (0.05), and a Z value of 1.96, based on this formula, the required sample size was determined to be 371 pharmacists for a 95% confidence level and a 5% margin of error, assuming a total population of 11,153 pharmacists in the UAE as per the UAE statistics for 2020 [57].

### 4.4. Ethical Considerations

Ethical approval was obtained from the research and ethics committee of Dubai Pharmacy College before the commencement of the study (Reference number: REC/FD/2023/02). All participants completed the survey voluntarily after providing informed consent to protect their privacy. No identifiers were collected from participants, and all data were kept completely anonymous.

### 4.5. Data Analysis

SPSS version 29 was employed for data analysis. Knowledge levels derived from the 25 questions were categorized as poor (less than 50%), moderate (50–80%), and good (more than 80%). Descriptive analysis and frequencies were utilized to assess knowledge distribution. Furthermore, a Chi-Square test with a significance level of *p* < 0.05 was conducted to explore associations between knowledge levels, demographics, and practice.

## 5. Limitations

This study provides valuable insights into pharmacists’ knowledge and practices regarding probiotics in the UAE, with strong internal consistency in the survey tool enhancing the reliability of the results. However, the use of convenience sampling and a higher concentration of participants from Dubai may limit the generalizability of the findings. Additionally, the focus on pharmacists excludes other healthcare professionals, and the use of an online survey may introduce social desirability and recall bias. Investigators recommend that future studies implement stricter control measures, such as supervised completion of surveys or the incorporation of mixed methods such as interviews or focus groups, to mitigate this bias and improve the reliability of the data collected. Despite these limitations, the study offers important contributions and lays the groundwork for further research.

## 6. Conclusions and Recommendations

In conclusion, this study provides valuable insights into pharmacists’ knowledge, perceptions, and practices regarding probiotics. Significant knowledge gaps were identified, particularly in the areas of cardiovascular health and the role of probiotics in mental health, such as treating depression. While pharmacists are generally aware of probiotics’ digestive health benefits, their understanding of broader applications remains limited. To address these gaps, targeted educational interventions are essential to improve pharmacists’ awareness and knowledge, allowing them to better guide patients on the diverse health benefits of probiotics, including immune support.

Public awareness campaigns are also necessary to enhance consumers’ understanding of probiotics’ advantages and promote their optimal use. Addressing economic challenges, such as the high cost and lack of probiotic insurance coverage, requires collaboration among stakeholders, including government entities and insurance companies. This collaboration is crucial for developing reimbursement strategies and advocating for insurance coverage, which can make probiotics more accessible and affordable to the public under economically practical conditions.

## Figures and Tables

**Table 1:** Demographic characteristics of participants.

Variable		N	%
Gender	Female	259	63.65%
	Male	148	36.4%
Educational Status	Diploma	115	28.3%
	Pharmacy Sciences Bachelors	190	46.7%
	Pharm D	68	16.7%
	Master’s degree	32	7.9%
	Doctorate or higher	2	0.5%
Country of highest education	UAE	211	51.8%
	Other Arab countries	24	5.7%
	Asia	136	33.5
	Others	36	8.8%
Type of pharmacy	Community Chain Pharmacy	212	52.1%
	Community Individual Pharmacy	82	20.1%
	Hospital pharmacy	113	27.8%
Location of the pharmacy	Abu Dhabi	13	3.2%
	Ajman	62	16.0%
	Dubai	259	63.6%
	Fujairah	2	0.5%
	Ras Al-Khaimah	3	0.7%
	Sharjah	65	16.0%
Number of prescriptions per day in your pharmacy	Less than 10	109	26.8%
	10–19	181	44.5%
	20–30	57	14.0%
	More than 30	60	14.7%
Years of experience	0–5	239	58.7%
	6–10	104	25.6%
	10–20	46	11.3%
	>20	18	4.4%
Age	22–30	262	64.4%
	30–40	105	25.8%
	40–50	35	8.6%
	>50	5	1.2%

**Table 2:** Knowledge levels of participants regarding probiotics and their various dosage forms.

Question		Correct Answer	Correct Answer *N* (%)	Wrong Answer *N* (%)
Indicate your answer for the following statements.	Probiotics consist of many strains of live microorganisms, each with specific health benefits.	Yes	388 (95.3%)	19 (4.7%)
	Yogurt is a natural source of beneficial bacteria.	Yes	390 (95.8%)	17 (4.2%)
	Bacteria are commonly used as probiotics.	Yes	358 (88.0%)	49 (12.0%)
	Lactobacillus acidophilus is used as a probiotic.	Yes	381 (93.6%)	26 (6.4%)
	Oral probiotics are the most effective dosage form.	Yes	280 (68.8%)	127 (31.2%)
Probiotics could be used to	Boost the human immune system.	Yes	371 (91.2%)	36 (8.8%)
	Treat gastrointestinal disorders (GERD, IBS, etc.).	Yes	303 (74.4%)	104 (25.6%)
	Reduce the risk of traveler’s diarrhea.	Yes	365 (89.7%)	42 (10.3%)
	Resolve allergy symptoms.	Yes	280 (68.8%)	127 (31.2%)
	Improve the respiratory tract system immunity.	Yes	198 (48.6%)	209 (51.4%)
	Reduce the recurrence of urinary tract infections.	Yes	354 (87.0%)	53 (13.0%)
	The overall health of the cardiovascular system.	Yes	242 (30.0%)	165 (70.0%)
	Alleviate depression symptoms.	Yes	122 (59.5%)	285 (40.5%)
	Improve oral health.	Yes	348 (85.5%)	59 (14.5%)
	Overall vaginal health.	Yes	360 (88.5%)	47 (11.5%)
Which type of probiotic may you recommend for patients using antibiotics to prevent antibiotic-associated diarrhea?	*Lactobacillus rhamnoses*	*Lactobacillus rhamnoses*	320 (78.6%)	87 (21.4%)
Available probiotics dosage forms in the pharma market	Capsules	Yes	376 (92.4%)	31 (7.6%)
	Tablets	Yes	250 (61.4%)	157 (38.6%)
	Syrup/elixir/drops	Yes	215 (52.8%)	192 (47.2%)
	Oral powder	Yes	318 (78.1%)	89 (21.9%)
	Topical cream/gel/spray	Yes	73 (17.9%)	334 (82.1%)
	Chewing gum	Yes	68 (16.7%)	339 (83.3%)
Available probiotic delivery systems in the pharma market	Nanoparticles	Yes	173 (42.5%)	234 (57.5%)
	Liposomes	No	232 (57.0%)	175 (43.0%)
	Microcapsules	Yes	215 (52.8%)	192 (47.2%)

**Table 3:** Participants’ perception of probiotic safety.

To What Extent Do You Agree with the Following Statement? (Agree, Strongly Agree, Neutral, Disagree, Strongly Disagree)	Strongly Disagree/ Disagree *N* (%)	Neutral *N* (%)	Strongly Agree/Agree *N* (%)	Mean ± S.D.
I would benefit from education or workshops related to the use of probiotics.	0 (0.0%)	30 (7.4%)	377 (92.6%)	2.93 ± 0.262
I think probiotics are safe to be used for pregnant women.	13 (3.2%)	77 (18.9%)	317 (77.9%)	2.75 ± 0.504
I recommend probiotics without any concerns.	30 (7.4%)	78 (19.2%)	299 (73.5%)	2.66 ± 0.610
I believe that the public is aware of the benefits and usage of probiotics.	57 (14.0%)	85 (20.9%)	265 (65.1%)	2.51 ± 0.729
I think probiotics are safe to be used for all ages.	92 (22.6%)	53 (13.0%)	262 (64.4%)	2.42 ± 0.835
I think probiotics are safe to be prescribed to immunocompromised patients.	149 (36.6%)	80 (19.7%)	178 (43.7%)	2.07 ± 0.895

**Table 4:** Participants’ practices regarding dispensing probiotics.

Questions		*N*	(%)
What is the frequency of selling the probiotics in your pharmacy (during the last year)?	Less than once a week.	38	9.3%
	Once a week	64	15.7%
	2–3 times a week	162	39.8%
	Daily	143	35.1%
How often do you prescribe probiotics to your patients to boost their immune system?	Never	3	0.7%
	Rarely	34	8.4%
	Sometimes	84	20.6%
	Often	179	44.0%
	Always	107	26.3%
When do you advise your patients to take probiotics concerning meals?	Before meals	268	65.8%
	After meals	87	21.4%
	Irrespective of meals	52	12.8%
What storage condition of the probiotic’s instruction do you emphasize to patients?	Store at room temperature	271	66.6%
	Store in the fridge	108	26.5%
	Others (depending on the type of probiotic)	21	4.3%
	Others	7	1.5%
When a patient reaches you asking about probiotics, what is your advice?	Prescribe a proper probiotic for the patient’s condition.	307	75.4%
	Ask the patient to follow up with the physician.	67	16.5%
	I recommend that the patient rely on natural resources for probiotics.	31	7.6%
	All	2	0.4%
When discussing probiotics with patients, what common misconceptions have you encountered?	Probiotics are only helpful for digestive issues	238	58.5%
	All probiotics are the same	145	35.6%
	Probiotics can replace a healthy diet	160	39.3%
	Probiotics are ineffective	177	43.5%
For which condition do you most frequently prescribe probiotics?	Gastrointestinal diseases	230	56.5%
	Vaginal infection	127	31.2%
	Allergies	34	8.4%
	All	13	2.6%
Which probiotic dosage forms are most requested by patients in your pharmacy?	Capsules/Tablets	249	61.2%
	Suppositories	9	2.2%
	Liquid dosage forms	49	12.0%
	Powder form	99	24.9%
	Semisolid dosage form	1	0.2%
What do you consider when recommending a specific probiotic dosage form to a patient?	The particular health condition the probiotic is intended for	322	79.1%
	Ease of administration	170	41.8%
	Potential for interactions with other medications	124	30.5%
	Financial constraints	129	31.7%

**Table 5:** Participants’ perceived barriers to the optimal use of probiotics.

What Do You Think of the Following Could Be a Barrier to the Optimum Use of Probiotics in the UAE?	Strongly Disagree/ Disagree *N* (%)	Neutral *N* (%)	Strongly Agree/ Agree *N* (%)	Mean ± S.D.
High cost of probiotics.	13 (3.2%)	42 (10.3%)	352 (86.5%)	2.83 ± 0.451
Lack of insurance coverage for probiotics.	18 (4.4%)	38 (9.3%)	351 (86.2%)	2.82 ± 0.488
Lack of recommendations by the physicians.	9 (2.2%)	54 (13.3%)	344 (84.5%)	2.82 ± 0.436
Poor patient compliance towards specific probiotic dosage forms.	32 (7.9%)	77 (18.9%)	298 (73.2%)	2.65 ± 0.620
Lack of demand by the public.	93 (22.9%)	51 (12.5%)	263 (64.6%)	2.42 ± 0.838

**Table 6:** Association between demographic characteristics and knowledge levels.

Demographic Characteristics		Knowledge of Probiotics			Chi-Square *p*-Value
		Good Knowledge (%)	Moderate Knowledge (%)	Poor Knowledge (%)	
Gender	Female	38 (14.7%)	207 (79.9%)	14 (5.4%)	0.044 *
	Male	33 (22.3%)	102 (68.9%)	13(8.8%)	
Education level	Diploma	5 (4.3%%)	102 (88.7%)	8 (7.0%)	0.001 *
	Doctorate or higher	1 (50.0%)	1 (50.0%)	0 (0.0%)	
	Master’s degree	10 (31.3%)	19 (59.4%)	3 (9.4%)	
	Pharmacy Sciences Bachelors	36 (18.9%)	142 (74.7%)	12 (6.3%)	
	PharmD	19 (27.9%)	45 (66.2%)	4 (5.9%)	
Type of pharmacy	Chain Community Pharmacy	4 (22.6%)	152 (71.7%)	12 (5.7)	0.013 *
	Individual Community Pharmacy	14 (17.1%)	6 (73.2%)	8 (9.8%)	
	Hospital pharmacy	9 (8.0%)	97 (85.8%)	7 (6.2%)	
Location of pharmacy	Abu Dhabi	2 (15.4%)	11 (%)	0 (0.0%)	0.011 *
	Ajman	20 (30.8%)	36 (%)	9 (%)	
	Dubai	35 (13.5%)	210 (%)	14 (%)	
	Fujairah	1 (50.0%)	1 (50.0%)	0 (%)	
	Ras Al-Khaimah	0 (0.0%)	3 (100.0%)	0 (0.0%)	
	Sharjah	13 (20.0%)	48 (73.8%)	4 (6.2%)	
Number of prescriptions per day in pharmacy	10–19 prescriptions	29 (16.0%)	146 (80.7%)	6 (3.3%)	0.072
	20–30 prescriptions	12 (21.2%)	38 (66.7%)	7 (12.3%)	
	Less than ten prescriptions	16 (14.7%)	82 (75.2%)	11 (10.1%)	
	More than 30 prescriptions	14 (23.3%)	43 (71.1%)	3 (5.0%)	
Sources of information	Books and magazines	43 (31.9%)	77 (57.0%)	15 (11.1%)	<0.001 *
	Media (TV, Radio)	33 (28.2%)	74 (63.2%)	10 (8.5%)	
	Social media	38 (15.5%)	196 (80.0%)	11 (4.5%)	
	Attending conferences and webinars	41 (15.8%)	205 (78.8%)	14 (5.4%)	
	Marketing campaigns of the probiotics manufacturers	36 (29.3%)	78 (63.4%)	9 (7.3%)	
	Workplace	50 (15.6%)	262 (81.6%)	9 (2.8%)	
	Journal articles	39 (28.1%)	88 (63.3%)	12 (8.6%)	
	Information leaflet	40 (25.0%)	104 (65.0%)	16 (10.0%)	
	Others	0 (0.0%)	7 (100.0%)	0 (0.0%)	
Years of experience	>20	3 (16.7%)	14 (77.8%)	1 (5.6%)	0.474
	0–5	34 (14.2%)	187 (78.2%)	18 (7.5%)	
	10–20	9 (19.6%)	35 (76.1%)	2 (4.3%)	
	6–10	25 (24.0%)	73 (70.2%)	6 (5.8%)	
Age	>50	0 (0.0%)	4 (80.0%)	1 (20.0%)	0.107
	20–30	36 (13.7%)	209 (79.8%)	17 (6.5%)	
	30–40	26 (24.8%)	72 (68.6%)	7 (5.7%)	
	40–50	9 (25.7%)	24 (68.6%)	2 (6.6%)	
Practice Section		Knowledge towards probiotics			Chi-square *p*-value
		Good knowledge (%)	Moderate knowledge (%)	Poor knowledge (%)	
How often do you prescribe probiotics to your patients to boost their immune system?	Always	36	6	3	<0.001 *
	Never	1	0	2	
	Often	16	160	3	
	Rarely	3	28	3	
	Sometimes	15	53	16	
What is the frequency of selling the probiotics in your pharmacy (during the last year)?	2–3 times a week	15	134	13	<0.001 *
	Daily	46	94	3	
	Less than once a week.	4	29	5	
	Once a week	6	52	6	
When a patient reaches you asking about probiotics, what is your advice?	Ask the patient to follow up with the physician.	14	42	12	<0.001 *
	Prescribe a proper probiotic for the patient’s condition.	53	244	11	
	I recommend that the patient rely on natural probiotic resources.	4	23	4	

* Indicates significant results at *p* level < 0.05.

**Table 7:** Multinomial logistic regression analysis of factors associated with moderate and good knowledge levels.

Demographics	Level	Moderate Knowledge			Good Knowledge		
		OR	CI	Sig	OR	CI	Sig
Gender	Male (ref.)						
	Female	1.884	(0.854–4.157)	0.117	1.069	(0.440–2.597)	0.882
Age category	40–50 (ref.)						
	>50	0.333	(0.240–4.595)	0.412	1.962	(1.500–2.500)	0.367
	30–40	0.857	(0.167–4.410)	0.854	0.825	(0.144–4.725)	0.829
	20–30	1.025	(0.223–4.707)	0.975	0.471	(0.092–2.419)	0.540
Highest education	PharmD (ref.)						
	Diploma	1.133	(0.325–3.957)	0.844	0.132	(0.028–0.622)	0.010
	Bachelors	1.052	(0.323–3.424)	0.933	0.632	(0.179–2.228)	0.475
	Master’s	0.563	(0.115–2.761)	0.479	0.702	(0.131–3.771)	0.680
	Doctorate	2.980	(1.500–5.234)	<0.001	7.07	(3.500–12.50)	<0.001
Experience category	6–10 (ref.)						
	0–5	0.854	(0.326–2.236)	0.748	0.453	(0.157–1.306)	0.143
	10–20	1.438	(0.276–7.491)	0.666	1.080	(0.184–6.356)	0.932
	>20	1.151	(0.128–10.3)	0.900	0.720	(0.063–8.197)	0.791
Type of pharmacy	Hospital (ref.)						
	Community Chain	0.914	(0.348–2.402)	0.855	7.411	(1.871–29.36)	0.004
	Community Individual	0.541	(0.187–1.569)	0.258	3.845	(0.733–20.15)	0.111
Prescriptions per day	>30 (ref.)						
	10–20	1.698	(0.407–7.073)	0.467	1.036	(0.225–4.762)	0.964
	20–30	0.379	(0.091–1.569)	0.181	0.367	(0.077–1.743)	0.207
	<10	0.520	(0.138–1.964)	0.335	0.312	(0.072–1.348)	0.119

## Data Availability

The data presented in this study are stored securely at Dubai Pharmacy College (DPC) and are available upon request from the corresponding author. Due to privacy and institutional regulations, the data are not publicly accessible.

## Appendix A. Questionnaire

Demographics
1. Gender: ○ Male ○ Female
2. Age (in years) Yr.
3. Your highest education ○ Diploma ○ Pharmacy Sciences Bachelors ○ PharmD ○ Master’s degree ○ Doctorate degree or higher
4. Country of highest education ○ UAE ○ Others (please specify)
5. Experience (in years): Yr.
6. Location of your pharmacy: ○ Abu Dhabi ○ Dubai ○ Ajman ○ Ras Al Khaimah ○ Sharjah ○ Umm Al Quwain ○ Fujairah
7. Type of the pharmacy: ○ Community pharmacy, Chain pharmacy ○ Community pharmacy, Individual pharmacy ○ Hospital pharmacy
8. Number of prescriptions per day in your pharmacy ○ Less than 10 prescriptions ○ 10–19 prescriptions ○ 20–30 prescriptions ○ More than 30 prescriptions
9. What is your sources of information on Probiotics (select all apply) ○ Books and magazines ○ Media (TV, Radio) ○ Social media ○ Attending conferences and webinars ○ Marketing campaigns of the probiotics manufacturers ○ At workplace ○ Journal articles ○ Information leaflet ○ Others
Knowledge
10. Indicate your answer for the following statements (yes, no, not sure) (a) Probiotics consist of live microorganisms that are helpful when consumed each with specific health benefits. (b) Yogurt is a natural source of beneficial bacteria. (c) Bacteria commonly used as probiotics. (d) Lactobacillus acidophilus is used as a probiotic. (e) Oral probiotics is the most effective dosage form
11. Indicate your answer for the following statements (yes, no, not sure) (a) Probiotics are used to boost the human immune system (b) Probiotics are used in treating gastrointestinal disorders (GERD, IBS) (c) Probiotics are used to reduce the risk of traveler’s diarrhea. (d) Probiotics are used to resolve allergy symptoms. (e) Probiotics are used to improve the respiratory tract system immunity. (f) Probiotics are used to reduce the recurrence of urinary tract infections. (g) Probiotics are used for the overall health of the cardiovascular system. (h) Probiotics are used to alleviate depression symptoms. (i) Probiotics are used to improve oral health. (j) Probiotics are used for overall vaginal health.
12. The available probiotics dosage forms in the pharma market are (select all apply) (a) Tablets (b) Capsules (c) Suppositories (d) Nasal spray (e) Semisolids (gel, creams) (f) Liquid dosage form. (g) Powder form. (h) Topical cream/gel/spray
13. Which type of probiotic may you recommend for patients using antibiotics to prevent antibiotic-associated diarrhea (a) *Lactobacillus rhamnoses* (b) *Saccharomyces cerevisiae* (c) *Streptococcus pyogenes* (d) *Escherichia coli*
14. The available probiotic delivery systems in the pharma market are (select all apply) (a) Nanoparticles (b) Liposomes (c) Microcapsules
Perception
15. To what extent do you agree with the following statement? (Agree, strongly agree, Neutral, Disagree, strongly disagree) (a) I think probiotics are safe to be used for all ages. (b) I think probiotics are safe to be used for pregnant and lactating women. (c) I think the probiotics oral dosage form is the most effective form. (d) I believe that the public is aware of the benefits and usage of probiotics. (e) I think that the public is using the probiotics optimally. (f) I would benefit from education or workshops related to the use of probiotics. (g) I recommend probiotics without any concerns.
Practice
16. What is the frequency of selling the probiotics in your pharmacy (during the last year) (a) Daily (b) 2–3 times a week (c) Once a week (d) Less than once a week.
17. How often you recommend your patients to use probiotics to boost their immune system: (a) Always (b) Often (c) Sometimes (d) Rarely (e) Never
18. When do you advise your patients to take probiotics in relation to meals? (a) Before meals (b) After meals (c) With meals (d) Irrespective of meals
19. What storage condition of the probiotic’s instruction do you emphasize to patients? (a) Keep them in the refrigerator. (b) Store them in a dry, cold area. (c) Leave them in direct sunlight. (d) Depends on the dosage form. (please specify)
20. When a patient reaches you asking about probiotics, what is your advice: (a) Prescribe a proper probiotic for the patient’s condition. (b) Ask the patient to follow up with the physician. (c) Recommend the patient to rely on natural resources of the probiotics.
21. When discussing probiotics with patients, what common misconceptions have you encountered? (Select all that apply.) (a) Probiotics are harmful to health (b) Probiotics are only helpful for digestive issues (c) All probiotics are the same (d) Probiotics can replace a healthy diet (e) Probiotics can cure any health problem (f) Probiotics are ineffective
22. For which condition you frequently prescribe Probiotics: (a) Gastrointestinal diseases. (b) Vaginal infection (c) Allergies (d) Other (please specify)
23. Which probiotic dosage forms are most requested by patients in your pharmacy? (a) Tablets (b) Capsules (c) Suppositories (d) Nasal sprays (e) Semisolids (gels, creams) (f) Liquid dosage forms (g) Powder form
24. What do you consider when recommending a specific probiotic dosage form to a patient? (Select all that apply.) (a) The specific health condition the probiotic is intended for (b) Ease of administration (c) Potential for interactions with other medications (d) financial constraints
25. For which patient groups do you recommend liquid probiotics? (Select all that apply.) (a) Infants and young children (b) Elderly patients (c) Patients with difficulty swallowing pills (d) Patients with acute gastrointestinal issues
Barriers
26. What do you think of the following could be a barrier to the optimum use of probiotics in the UAE. (a) Lack of recommendations by the physicians. (b) Lack of demand by the public. (c) High cost of probiotics (d) Poor patient compliance towards specific probiotic dosage forms (e) Lack of insurance coverage of probiotics
